# Designing contrasts for rapid, simultaneous parameter quantification and flow visualization with quantitative transient-state imaging

**DOI:** 10.1038/s41598-019-44832-w

**Published:** 2019-06-11

**Authors:** Pedro A. Gómez, Miguel Molina-Romero, Guido Buonincontri, Marion I. Menzel, Bjoern H. Menze

**Affiliations:** 1 Technical University of Munich, Munich, Germany; 2 GE Healthcare, Munich, Germany; 3 Imago7 Foundation, Pisa, Italy; 4 IRCCS Stella Maris, Pisa, Italy

**Keywords:** Diagnostic markers, Biomedical engineering

## Abstract

Magnetic resonance imaging (MRI) has evolved into an outstandingly versatile diagnostic modality, as it has the ability to non-invasively produce detailed information on a tissue’s structure and function. Complementary data is normally obtained in separate measurements, either as contrast-weighted images, which are fast and simple to acquire, or as quantitative parametric maps, which offer an absolute quantification of underlying biophysical effects, such as relaxation times or flow. Here, we demonstrate how to acquire and reconstruct data in a transient-state with a dual purpose: 1 – to generate contrast-weighted images that can be adjusted to emphasise clinically relevant image biomarkers; exemplified with signal modulation according to flow to obtain angiography information, and 2 – to simultaneously infer multiple quantitative parameters with a single, highly accelerated acquisition. This is achieved by introducing three novel elements: a model that accounts for flowing blood, a method for sequence design using smooth flip angle excitation patterns that incorporates both parameter encoding and signal contrast, and the reconstruction of temporally resolved contrast-weighted images. From these images we simultaneously obtain angiography projections and multiple quantitative maps. By doing so, we increase the amount of clinically relevant data without adding measurement time, creating new dimensions for biomarker exploration and adding value to MR examinations for patients and clinicians alike.

## Introduction

### MR contrasts versus parameter quantification

Since it’s inception^1^, magnetic resonance imaging (MRI) has become a cornerstone technology in diagnostic imaging. Through careful calibration of magnetic gradients and pulses, MR scanners provide wide-ranging physiological and anatomical details of imaged objects. The most common representation of this information takes the form of contrast-weighted images, where data is qualitatively weighted by the longitudinal relaxation time (T1), the transverse relaxation time (T2), or the proton density (PD). However, contrast-weighted images are subjective: the expertise of radiologists plays a key role in their evaluation for disease diagnosis and monitoring. To this end, advanced mapping techniques have been developed – all based on series of weighted acquisitions – that offer means for an absolute quantification of biophysical phenomena, such as tissue relaxation time or displacement velocity, and that help in increasing accuracy and reproducibility of diagnostic information^2^. Notwithstanding, it is contrast, not quantification, which has allowed MRI to thrive as one of the most important diagnostic modalities.

### Advantages of qualitative MR contrasts

The fundamental reason for the success of contrast – i.e. relative local differences in qualitative images – over quantification can be attributed to the simpler process of signal encoding: whereas contrast-weighted MRI merely *accounts* for physical effects interacting with the acquired signal to produce clinically relevant images, quantitative MRI aims at *encoding* for these. This adds complexity to the acquisition: First, additional samples need to be acquired along multiple encoding dimensions, consequently increasing scan times, sometimes beyond clinical acceptance. Second, most quantitative approaches probe only a single or a few parameters at a time, requiring lengthy serial acquisitions if multiparametric information is required. Finally, quantitative parameters are estimated by enforcing consistency with a biophysical model, which, by definition, is subject to limitations.

As an example, MR angiographic scans are fast and simple to acquire, and even though they do not quantify the velocity or direction of flowing blood in an absolute fashion, they represent an indispensable biomarker in the diagnosis of strokes^3^, stenosis^4^, and other vascular diseases. 4D flow MRI^5^, the quantitative counterpart of MR angiography, is significantly more involved: it requires multiple directional velocity encoding gradients to capture time-varying velocity vectors and a corresponding high-dimensional, spatiotemporal biophysical model to accurately measure and visualize complex blood flow patterns. While promising, the cost in terms of increased acquisition time and the uncertainty of the resulting flow quantification still prevents the widespread clinical adoption of 4D flow techniques^6^. Thus, with flow – as with other quantitative techniques – the current provider of clinical value remains contrast rather than quantification.

### The advantage and the shortcomings of novel quantitative sequences

The recent advent of fast multiparametric mapping methods offers new directions for challenging the longstanding dominance of contrast-weighted imaging biomarkers. Novel techniques such as MR fingerprinting (MRF)^7^, synthetic MRI^8^, and others^9–13^ promise to ease the clinical implementation of quantitative imaging techniques by developing sequences to quickly deliver multiparametric information with a single scan. Recent advances offer high-resolution maps with ground-breaking speed^14^, creating new clinical opportunities for accelerated, quantitative mapping. These methods have already been extended to consider the effects of blood flow by deriving vascular parameters after contrast injection^15,16^, computing velocity scalars^17^ or vectors in phantom studies^18^. However, such quantitative techniques share a common drawback: they are designed to exclusively provide the information derived from the biophysical model, and since quantitative information obtained from simplified modelling is subject to limitations, could disregard valuable diagnostic information.

For instance, Cao *et al*.^17^ describe a fingerprinting technique to derive flow perpendicular to the slice by simulating different velocities in an exhaustive dictionary. This model-based velocity mapping produces scalar velocity values together with T1, T2 and proton density. However, the acquisition does not explicitly encode for flow with e.g. velocity encoding gradients, resulting in flow estimates appearing exclusively in large vessels without capturing motion phenomena in smaller or slowing flowing vessels. Flassbeck *et al*.^18^ do introduce velocity encoding gradients, demonstrating quantification of T1 and T2 of stationary tissues together with a 3D velocity vector within flowing vessels. This promising approach requires nonetheless three minutes per slice and works under the simplifying assumption of a regular flow pattern, neither of which are adequate for *in vivo* applications. Both techniques deliver limited and uncertain quantitative velocity information and by overlooking the underlying process of image formation, neglect the clinical value provided by qualitative contrasts produced by flowing spins.

### Our contribution: designing qualitative contrasts with a quantitative sequence

Here, instead of limiting the acquisition to obtain exclusively quantitative parameters, we propose to reconstruct spatially resolved temporal signals that accommodate additional sources of information, opening the door to new possibilities for data analysis and biomarker exploration. Our solution is inspired by the promising advances shown by MRF but removes its two signature features – namely a pseudorandom acquisition and the matching of signals to a precomputed dictionary – and replaces them with a sequence design and parameter inference framework to achieve a dual purpose: 1 – create contrasts for clinically relevant image biomarkers, and 2 – infer multiple quantitative maps – both simultaneously with a single scan. We term our method ‘quantitative transient-state imaging’ and achieve our dual purpose by combining three novel elements:

1. First, our solution builds a ***transient-state model*** based on the latest multiparametric advances for quantitative encoding, while also ***considering motion phenomena*** that perturb transient signals during acquisition time.
2. Second, it offers a ***design framework for extracting diagnostically relevant contrasts*** from these physiological effects during parameter encoding with smooth excitation patterns.
3. Third, it relies on a ***4D reconstruction to create hundreds of contrast-weighted images*** from which relevant image biomarkers can be obtained ***alongside the quantitative parameters*** of the Bloch equations. Moreover, the parameters are inferred probabilistically from the model in the transient-state, resulting in additional estimates of parametric uncertainty.

The first component of our solution relies on transient-state imaging, where, different to steady-state approaches, signals evolve dynamically throughout data acquisition. This dynamic signal evolution produces imaging artifacts when measurements from different repetitions are combined, limiting efforts to create contrast-weighted images^19,20^ or map parameters^21^ in the transient-state. Nonetheless, the transient signal dependence on T1 and T2 is well characterised^22,23^ and recursive simulations can be used to describe any pattern of temporally varying magnetisation vectors. These simulations, in turn, enable the reduction of imaging artifacts, as they can be used to impose constraints during data reconstruction. Alternatively, they also enable direct parameter quantification by matching simulations to the acquired data, as shown in MRF. Our solution also builds on such recursive simulations, namely on the Extended Phase Graph (EPG)^24,25^ formalism, introducing an additional term to account for flowing spins during data acquisition.

The second component of our solution concerns the design of a transient-state sequence that enables both the creation of clinically relevant contrasts and efficient parameter encoding. Here, we follow the hypothesis of recent optimisation work establishing that pseudo-random acquisitions do not allow for optimal quantification performance^26–29^. We approach the design problem using Bayesian decision theory^30,31^, where we use the introduced transient-state signal model and prior parameter distributions to find a scheme that maximises contrasts with optimal parameter encoding.

The third and final component of our solution proposes a 4D spatiotemporal reconstruction to obtain time-resolved contrast-weighted images. In order to achieve this, we rely on non-Cartesian *k*-space sampling and advanced reconstruction techniques inspired from the theory of compressed sensing^32^. By doing so, we take a similar line to other techniques which have proved successful for different kinds of dynamic MRI data^33–36^, including MRF^26,37–39^; whereas we introduce a local low rank regularization on 4D spatiotemporal image neighbourhoods.

This 4D reconstruction enables different manners of processing the data. For instance, we use Bayesian inference to estimate parametric maps and their associated uncertainty. In addition to providing quantitative PD, T1 and T2 maps of the brain, QTI also returns an image time series which is shown to provide complementary biophysical information. In this report, the latter is exploited to visualize – and emphasise – specific physiological features like blood volumes or tissue types. In this way, we maintain the advantages of clinically successful contrast-weighted ‘qualitative’ imaging using a sequence that is intrinsically quantitative in nature.

We illustrate our flow-resolved QTI application by modulating signals in the transient-state to encode for relaxation times while simultaneously accounting for the effects of flowing blood. In this manner, we can obtain – on the one hand – multiple contrast-weighted images, including specifically designed contrasts that carry the information of MR angiography images; and – on the other – fast, high-resolution, and accurate measurements of the encoded parameters. By doing so, we add vasculature biomarkers without increasing scan times and offer novel prospects for precision medicine applications, such as designing disease- and biomarker-specific imaging sequences.

## Materials and Methods

### Formalising flow dynamics in the transient-state

A strategy to encode for multiple tissue properties, demonstrated in MRF^7^, is to prevent the formation of a steady-state by continuously changing acquisition parameters. In this transient-state, magnetization evolves dynamically in time and, following EPG theory, can be described as the sum of Fourier configuration states with a state matrix ${\boldsymbol{\Omega }}\in {{\mathbb{C}}}^{3\times k}$

1 $${\boldsymbol{\Omega }}=[\begin{array}{ccc}{F}_{0} & \ldots  & {F}_{k}\\ {F}_{0}^{\ast } & \ldots  & {F}_{k}^{\ast }\\ {Z}_{0} & \ldots  & {Z}_{k}\end{array}].$$

In this state matrix, *F*_*k*_ and ${F}_{k}^{\ast }$ represent dephasing and rephasing transverse magnetization components and *Z*_*k*_ is the longitudinal magnetization at different dephasing states *k*. Per EPG theory, all MRI sequences can be described by a temporally varying state matrix **Ω**_*t*_(·), where linear operators modify **Ω**_*t*_ to account for effects such as tissue relaxation, gradient dephasing, motion or radiofrequency excitation (a detailed EPG review can be found in Weigel *et al*.^24^). Thereafter, the progression of the state matrix may be determined recursively:

2 $${{\boldsymbol{\Omega }}}_{t}{\boldsymbol{(}}\eta ;\theta {\boldsymbol{)}}={{\bf{g}}}_{t}{\boldsymbol{(}}\eta ;\theta {\boldsymbol{)}}{{\boldsymbol{\Omega }}}_{t-1}{\boldsymbol{(}}\eta ;\theta {\boldsymbol{)}}.$$

In Eq. 2, the state matrix **Ω**_*t*_(·) at time *t* is defined by the action of the linear operators **g**_*t*_(·) on the magnetization components at time *t* − 1. These operators, in turn, are a function of two distinct sets: *η*, the temporally varying variables one determines in designing an acquisition scheme, such as the flip angle or repetition time; and *θ*, the spatially varying biophysical parameters we wish to infer. Following Weigel’s notation, and neglecting incoherent motion, magnetisation transfer, and other effects, the operator **g**_*t*_(·) takes the form:

3 $${{\bf{g}}}_{t}(\eta ;\theta )={\bf{E}}(\theta ){{\bf{S}}}_{t}(\eta ){{\bf{J}}}_{t}(\eta ;\theta ){{\bf{T}}}_{t}(\eta ),$$

where **E**(*θ*) characterises T1 and T2 relaxation, **S**_*t*_(*η*) accounts for the presence of unbalanced gradient waveforms between excitations, **J**_*t*_(*η*; *θ*) is the flow operator, and **T**_*t*_(*η*) represents the action of radiofrequency excitation pulses on the configuration matrix. While *η* changes over time, we’ve decided to identify time dependence directly on the operators, e.g. **J**_*t*_(*η*; *θ*), to avoid adding additional dependencies to the variable *η*.

In this work, we propose to account for motion by considering spins flowing into the imaging slice. To do so, we rely on the concept of washout introduced by Axel^40^, where spins tagged by radiofrequency pulses wash out of the slice to be replaced with fresh magnetization entering the slice. Assuming a constant velocity over time and space, spins leave the slice at a linear rate of

4 $${h}_{t}(\eta ;\theta )=1-t\cdot (v/d),\,t\le d/v,$$

where the velocity *v* belongs to *θ* and the slice thickness *d* to *η*. Considering only perpendicular motion, *h*_*t*_(*η*; *θ*) acts on by **Ω**_*t*_(·) by decreasing all configuration states where *k* > 0, while introducing unsaturated magnetization to the longitudinal component *Z*_0_. We thus obtain a new formulation for the evolution of the state matrix:

5 $${{\boldsymbol{\Omega }}}_{t}(\eta ;\theta )={h}_{t}(\eta ;\theta ){{\bf{g}}}_{t}(\eta ;\theta ){{\boldsymbol{\Omega }}}_{t-1}(\eta ;\theta )+(1-{h}_{t}(\eta ;\theta ))(\begin{array}{ccc}0 & \ldots  & 0\\ 0 & \ldots  & 0\\ 1 & \ldots  & 0\end{array}).$$

When there is no flow, i.e. *h*_*t*_(·) = 1, spins become saturated from the continuous application of radiofrequency pulses and follow the dynamics dictated by the choice of acquisition parameters *η*. In the presence of flow, unsaturated spins in every repetition wash into the imaging slice, perturbing the signal throughout the acquisition. Note that *h*_*t*_(·) accounts exclusively for flow perpendicular to the imaging slice and is agnostic to the direction of flow and can therefore be used to capture both venous and arterial flow. Finally, according to EPG, the formation of echoes is represented by the *F*_0_ state. Hence, the complex measured signal over time can be directly defined as *f*_*t*_(*η*; *θ*) = *ρF*_0_(*t*), where *ρ* represents a scaling factor related to the proton density. We take advantage of this model to design a sequence by modifying *η* to optimally encode for *θ* while allowing signals to evolve towards clinically relevant tissue contrasts.

### Bayesian experimental design for contrast maximisation with optimal encoding

Even though it is possible to use random and arbitrary patterns of *η* to encode for *θ*, recent optimisation work has demonstrated that experimental efficiency can be significantly increased by designing the acquisition scheme to satisfy specific criteria^26–29^. Here, experimental design is guided by Bayesian decision theory^30,31^, where we account for prior probability distributions of parameters to determine the acquisition scheme.

The goal of Bayesian experimental design is thus to identify the optimal set of design variables *η* that maximises the encoding of the biophysical parameters of interest *θ* for a given prior distribution of these parameters. This is achieved by selecting the design variables that maximise the information gain of the parameters. Assuming a Gaussian noise model, the information gain for a particular *θ* set can be represented as a utility functional equivalent to the determinant of the Fisher information matrix:

6 $$u(\eta ;\theta )=det\sum _{t}(\begin{array}{ccc}\frac{{{\rm{\partial }}}^{2}{f}_{t}}{{\rm{\partial }}{\theta }_{1}^{2}} & \ldots  & \frac{{{\rm{\partial }}}^{2}{f}_{t}}{{\rm{\partial }}{\theta }_{1}{\theta }_{N}}\\ \vdots  & \ddots  & \vdots \\ \frac{{{\rm{\partial }}}^{2}{f}_{t}}{{\rm{\partial }}{\theta }_{N}{\theta }_{1}} & \ldots  & \frac{{{\rm{\partial }}}^{2}{f}_{t}}{{\rm{\partial }}{\theta }_{N}^{2}}\end{array}).$$

Note that a Gaussian noise model is a simplifying assumption, as *f*_*t*_(*η*; *θ*) is acquired as a complex vector and therefore its magnitude follows a Rician noise distribution. Nonetheless, this Bayesian framework resulted advantageous despite the simplifying assumption, as it allows for the definition of the utility functional, and in a Bayesian manner, the overall utility can be determined by marginalising the functional over a prior *π*(*θ*):

7 $$U(\eta )={\int }_{\theta }\mathrm{log}\,u(\eta ;\theta )\pi (\theta )d\theta .$$

Finally, the contrast created by the mean values of the different tissue classes in the prior can be incorporated into a final cost function:

8 $$C(\eta )=-\,((1-\sum {\mu }_{c})U(\eta )+{\sum }_{c=1}^{C}{\mu }_{c}{max}_{{f}_{t}}|{f}_{t}(\eta ;{\theta }_{c})-{f}_{t}(\eta ;{\theta }_{d})|{\rm{\forall }}\,d\ne c)$$

In Eq. 8, the cost function *C*(*η*) is minimised when the overall utility *U*(*η*) is maximised and the contrast for each individual tissue class *c* is maximised with respect to all other tissue classes, where *μ*_*c*_ controls the contribution of each tissue class and the overall weighting of utility maximisation versus contrast maximisation. Since Eq. 8 is non-convex, global optimisation techniques, such as genetic algorithms, are required. If the dimensionality of the design space is small enough, even exhaustive searches can be performed, as the optimal design variables *η* need to be determined only once and the optimisation can be performed offline.

**Figure 1:**
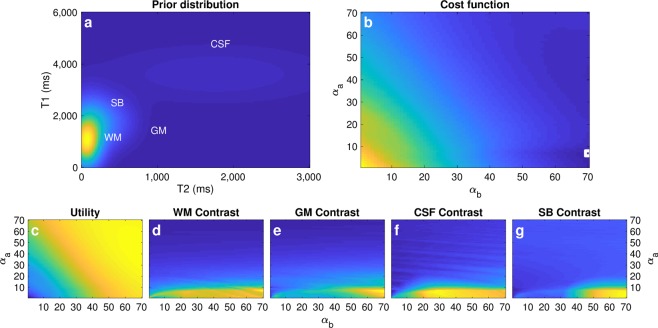
Constrained Bayesian experimental design. (**a**) Parameter space with prior probability distributions of each tissue class: grey matter (GM), white matter (WM), cerebrospinal fluid (CSF), and stationary blood (SB) (**b**) Visualization of optimization landscape with respect to the initial (α_a_) and final (α_b_) flip angle. The cost function reaches a minimum when α_a_/α_b_ is 7/70 degrees (white square). (**c**–**g)** Contribution of the individual terms to the final cost function. Whereas the utility is larger for larger initial and final flip angles, maximisation of contrast is achieved with small initial flip angles (α_a_ < 10 degrees). Weighting of each individual cost function term is application specific and can result in different designs for distinct use cases.

We assumed a broad Gaussian distribution of four tissue classes with T1/T2 values: 1,450/85 ms for grey matter (GM), 900/60 ms for white matter (WM), 3600/1750 for cerebrospinal fluid (CSF), and 1740/275 for stationary blood (Fig. 1a). We started by inverting the magnetization with an inversion pulse and found the optimal flip angle design *η* by setting two control points (minimum and maximum flip angle) and solving for Eq. 8 using a normalized *μ*_*c*_ = [0.05, 0.05, 0.1, 0.3] for GM, WM, CSF and SB respectively. To derive a computationally tractable solution in parameter space, we followed the approach described in Owen *et al*.^31^, while we additionally visualize the optimization landscape in Fig. 1b–g. The mean tissue values were only considered for the stationary case (*h*_*t*_(·) = 1), as in the domain of linear flip angle variations, the contrast for stationary blood after the inversion acts as a lower bound for flowing blood after inversion (Supplementary Fig. 1). Certainly, a smooth excitation pattern with two control points simplifies the optimization but might not be the globally optimal scheme. We also used the same design framework to find other higher-order flip angle patterns with more control points, consistently finding similar patterns of increasing flip angles (Supplementary Fig. 2).

**Figure 2:**
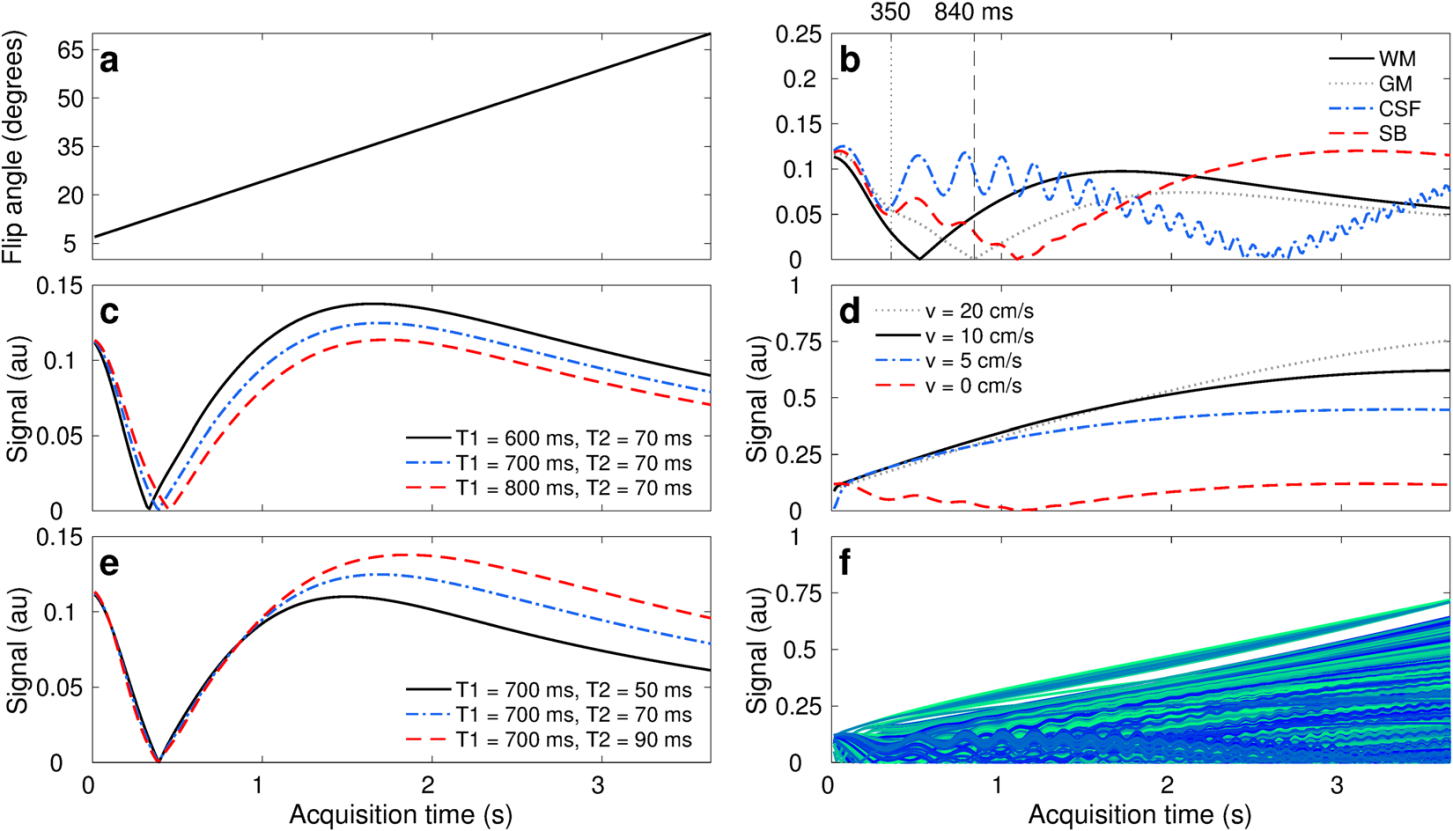
Parameter encoding scheme and signal dynamics in the presence of flow. (**a**) Encoding strategy given by linear flip angles variations. (**b)** Signal evolution for each tissue class: grey matter (GM), white matter (WM), cerebrospinal fluid (CSF), and stationary blood (SB). The sequence has been designed to have distinct signal evolutions for each class; where SB contrast discriminates well from the others towards the end of the sequence. Our proposed reconstruction framework also enables the visualisation of tissue dynamics through time, where e.g. WM/GM tissue contrasts at 350 and 840 ms are inverted as they recover from the inversion pulse with different T1 relaxation times. (**c**,**e**) Representation of parameter encoding with transient-state signal evolutions. Tissues with shorter T1 times experience their inversion first in time (solid black line in **c**) whereas tissues with longer T2 times produce larger stimulated echoes and thus less signal decay (dashed red line in **e**). (**d)** Hyperintensities due to fast flowing spins with 2 mm slice thickness. The thinner the imaging slice or the faster the flow, the more pronounced flow effects become. (**f**) Cohort of signals obtained by sampling the prior probability distribution of stationary and non-stationary tissues. As signals exhibit correlation in space and time, lower dimensional constraints can be imposed for image reconstruction. The proposed design scheme satisfies maximal contrast conditions and optimal parameter encoding in 3.66 seconds per imaging slice.

After an initial inversion, the obtained design is a flip angle ramp with initial/final flip angles of 7/70 degrees (Fig. 2a), 260 repetitions, repetition and echo times TE/TR = 2/14, 2 mm thick slices, and gradient dephasing per repetition (Supplementary Fig. 3). The first portion of this acquisition scheme encodes principally for T1, relying on the inversion pulse followed by small flip angles. The latter segment provides T2 encoding, as higher flip angles allow for the formation of stimulated echoes. Thereafter, tissues with shorter T1 times will invert first (Fig. 2c), while tissues with shorter T2 times will experience larger signal decays caused by smaller stimulated echoes in subsequent excitations (Fig. 2d). Moreover, contrast maximisation for SB towards the end of the acquisition combined with the thin imaging slices amplify flow effects, from which we recover angiography information. Preliminary sequence design work to simultaneously encode for parameters and recover angiography information can be found in the supplementary material of the first author’s doctoral thesis *Accelerating Quantitative Magnetic Resonance Imaging*^41^.

Finally, the proposed design results in smooth signal variations with respect to *θ*, leading to a certain level of correlation between an ensemble of multiple transient-state signals (Fig. 2f). That is, signal dynamics are governed by the exponential nature of the Bloch equations and consequently tissues with neighbouring parameters will exhibit akin signal evolutions, while tissues with distinct parameter combinations will evolve differently throughout the acquisition. We explicitly rely on the correlation between signals to reconstruct the spatiotemporal image space via lower dimensional signal constraints.

### 4D spatiotemporal image reconstruction

As the signal evolves in the transient-state, we acquire undersampled spatial data with a spiral arm in every repetition (Supplementary Fig. 3), since acquiring the full temporal signal for every 3D voxel in the image would increase the scan time to several hours^42^. We then use iterative reconstruction algorithms to obtain images from the undersampled measurements.

Specifically, we consider both the Fourier encoding of spatial signals and the temporal spin dynamics to formulate the image reconstruction problem^34,41,43^. With this formulation, an image *x*_*t*_ at time *t* is linearly encoded into the acquired measurements *y*_*t*_ with the operator *E*_*t*_:

9 $${y}_{t}={E}_{t}{x}_{t}.$$

The operator *E*_*t *_consists of three terms: *E*_*t*_ = *U*_*t*_*FS*. *U*_*t*_ is the undersampled *k*-space acquisition trajectory, corresponding to an arm of a spiral waveform designed with time-optimal gradients^44^. *F* represents the non-uniform fast Fourier transform^45^ and *S* are the coil sensitivities, obtained via adaptive coil combination of the acquired data^46^. By additionally incorporating a temporal subspace projection^26,34,38^ operation into the encoding operator, we used the alternating direction method of multipliers^34,38^ to reconstruct ten subspace images, where image regularization was achieved by applying a local low rank threshold on spatiotemporal images patches^34,39^ of dimension 8 × 8 × 8 × 260. The full spatiotemporal data space can be subsequently computed by linearly projecting the subspace images back to the temporal space^41^.

### Angiographic projections and probabilistic parameter inference

Obtaining spatially resolved MR signals allows to us to perform different forms of analysis on the reconstructed data. On the one hand, a qualitative combination of different time frames enables the creation of angiography images. This combination is achieved by summing the magnitude images of chosen frames and subsequently performing a maximum intensity projection. This qualitative assessment allows for a visualization of imaging biomarkers – such as those given by angiography – that may not be readily available in the quantitative parametric maps.

On the other hand, a quantitative analysis of the data allows us to simultaneously infer PD, T1, and T2. An important distinction to previous work in parametric mapping is that we do not perform inference by exhaustive dictionary matching but use Bayesian inference for uncertainty quantification and propagation. We used Π4U^47^, a high-performance computing framework based on Transitional Markov Chain Monte Carlo sampling, to obtain the posterior probability density function *p*(*θ*|*x*_*t*_, *f*_*t*_) of the parameters from the signal model *f*_*t*_ and the data *x*_*t*_:

10 $$p(\theta |{x}_{t},{f}_{t})=\frac{p({x}_{t}|\theta ,{f}_{t})\pi (\theta )}{p({x}_{t}|{f}_{t})}.$$

In Eq. 10 *p*(*x*_*t*_|*f*_*t*_) is the evidence of the model, *p*(*x*_*t*_|*θ*, *f*_*t*_) determines the probability of observing the data given the model, and *π*(*θ*) is the prior probability distribution. For Bayesian inference, unlike Bayesian experimental design, we assume a uniform prior in parameter space to avoid biasing parameter quantification. From *p*(*θ*|*x*_*t*_, *f*_*t*_) we can compute the expected value of each parameter in the model, their maximum likelihood, and related uncertainty. The introduction of uncertainty quantification with the probabilistic inversion of the transient-sate model provides complementary voxel-wise information on the correspondence between the reconstructed signals and the estimated maps. Uncertainty quantification also gives an indication of consistency of the measurements regarding the model, resulting in additional feedback on the encoding capabilities of the sequence with respect to each individual parameter.

### Data acquisition and analysis

Important benchmarks for parametric mapping techniques are the accuracy and efficiency of the obtained measurements. Thereafter, we obtained reference measurements from agar phantoms using gold-standard T1 and T2 techniques and compared them against QTI and the first MRF implementation with unbalanced gradients^48^ which has been used in multiple subsequent works^38,42,49–51^, as it is robust to artifacts and has been extensively validated for reproducibility^52^. We also shortened the MRF acquisition from 1000 repetitions to 280, resulting in the same scan time as QTI and refer to this shortened version as MRF*.

We performed an analysis of accuracy and efficiency of each method using agar phantoms with distinct T1 and T2 values^53^. The gold standard was obtained with inversion recovery and multi-echo spin echo acquisitions for T1/T2 quantification. The measurements were done with 22.5 × 22.5 cm^2^ field of view, 128 × 128 matrix size, 5 mm thickness, TR = 12,000 ms, TI = {2700, 680, 300, 1950, 1320, 3745, 815, 3490, 2000, 1700, 950, 550, 3600, 2600, 3100, 3900, 3400, 3230, 2350, 1800, 50, 2850, 170, 430, 2210, 2980, 1580, 1000, 2470, 1200, 1500} ms, and TE = {20, 300, 100, 600, 75, 200, 35, 400, 150, 50} ms. Both the TI and TE were acquired in this random order to minimise magnetic drift and temperature effects. We obtained 10 independent measurements for QTI, MRF, and MRF*, the truncated version of MRF to 3.66 seconds (280 repetitions). Statistical analysis was performed with 5,000 total samples per tube, obtained over the 10 measurements. The concordance correlation coefficient and efficiency were calculated following the first MRF publications^7,48^ considering the following time for each sequence: 3.66 s for QTI and MRF* and 13.13 s for MRF (0.02 s inversion pulse plus 13.11 readout).

We also scanned 112, 2 mm slices of two healthy volunteers (male, 28 and 26 years) with the proposed method (acquisition time = 6:48) and with a 3D axial time of flight angiography sequence with TE/TR = 2.1/24, 22 × 22 cm^2^ field of view, 256 × 256 matrix, acceleration factor 2 in phase, and acquisition time = 10:48. The *in vivo* scans were conducted complying with the German Act on Medical Devices without requiring IRB approval, as our methodological work does not amount to a clinical investigation and the volunteers (experts in MRI and researchers in our team) where fully aware of potential risks. Written informed consent was obtained for each volunteer and all experiments were performed on a machine that has met all safety and functionality requirements for its intended purpose. We acquired all data with a variable density spiral waveform with 89 total interleaves, requiring 18 of these for the centre of *k*-space. Data was acquired with a 22.5 × 22.5 cm^2^ field of view and 1.2 mm^2^ in-plane resolution. We rotated the waveforms with the golden angle to increase sampling incoherence^54,55^. Experiments were performed using a 3 T 750w scanner (GE Healthcare, Milwaukee, WI), with a 12-channel head coil.

## Results

### Phantom study

**Figure 3:**
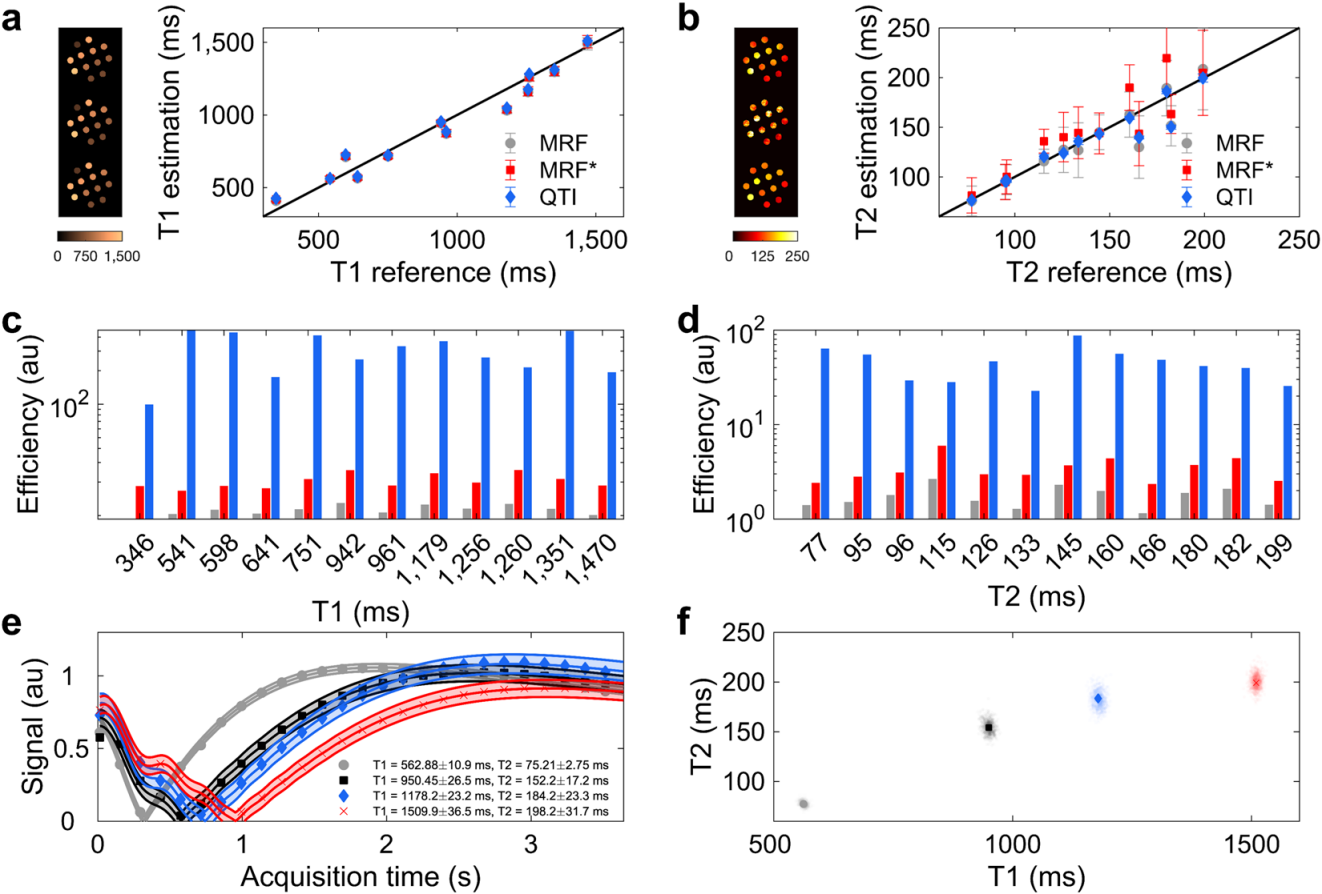
Phantom validation and uncertainty quantification. **(a**,**b**) Measurement accuracy with respect to the gold standard. Measurements evidence mean ± standard deviation from 5,000 voxels for each tube obtained from regions of interest from 10 independent measurements. MRF* refers to MRF truncated to a 3.66 second acquisition and the images on the left show, from top to bottom, the estimated parametric maps in the phantom for MRF, MRF*, and QTI. (**c**,**d**) Efficiency of each individual method, computed from the precision and acquisition time. (**e**) Transient-state signal evolution for four representative voxels in the phantom. The points represent measured data and the lines are the simulated signal evolutions which best fit the data along with their resultant uncertainty. Here, we can observe a trend of increased uncertainty with increasing T1/T2 values. (**f)** Posterior probability density function of the four representative voxels in parameter space and maximum likelihood estimation. The posterior density function becomes broader as the parameters increase, showing again the trend of increased uncertainty with increasing parameter values. This also gives an indication of parameter encoding: the lower the uncertainty, the better the corresponding parameters are encoded into the signal. QTI is thus an accurate and efficient method for multiparametric estimation and uncertainty quantification.

Figure 3a,b show that both QTI and MRF are in good agreement to the reference, achieving T1/T2 correlation coefficients of 0.9784/0.8788 and 0.9805/0.9497, respectively. MRF*, however, is only accurate for T1 measurements, while it deviates for T2, showing a T1/T2 correlation of 0.9775/0.5695. Figure 3c,d compares the efficiency of each method, defined as precision divided by the root of the acquisition time, where the increase in QTI efficiency can be attributed to both factors: increased precision in reduced time. Finally, Fig. 3e,f show the results of parameter estimation and uncertainty quantification using Π4U for Bayesian inference^47^. Table 1 compares QTI against other 2D MRF variants, where it can be observed that QTI obtains information from marginally smaller voxels and with similar speed as the optimized version of Assländer *et al*.^14^, with the added benefit of parameter uncertainty quantification and creation of angiography images. Also, while the examples shown here do not account for inhomogeneous transmit fields via B1+ mapping^42,56^, our inference framework enables the integration of separately acquired B1+ maps for parameter quantification. Henceforth, by relying on other fast B1+ mapping techniques^57^, the techniques shown here could be used at multiple field strengths with high efficiency. To ensure consistency with these previous MRF variants, no other physical effects such as B0 inhomogeneities, diffusion, or magnetisation transfer were considered in our analysis.

**Table 1:** Resolution, scan time, and capabilities of QTI versus different 2D MRF variants.

	QTI	MRF			
		Ma *et al*.^7^	Jiang *et al*.^48^	Cloos *et al*.^69^	Assländer *et al*.^14^
Resolution (mm^3^)	1.2 × 1.2 × 2	2.3 × 2.3 × 5	1.17 × 1.17 × 5	1.4 × 1.4 × 5	1 × 1 × 3
Scan time per slice (s)	3.66	12	13	7–21	3.80
Clinical parameters	T1, T2, PD	T1, T2, PD	T1, T2, PD	T1, T2, PD	T1, T2, PD
B1^+^ mapping	No	No	No	Yes	No
Parameter uncertainty	Yes	No	No	No	No
Angiography	Yes	No	No	No	No

### *In vivo* evaluation

**Figure 4:**
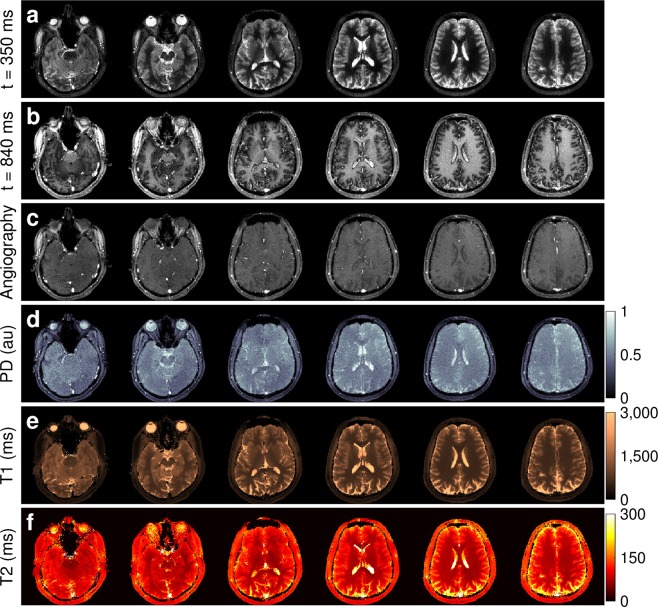
Simultaneous contrast-weighted imaging, angiography, and multiparametric mapping. (**a**,**b**) The proposed reconstruction framework allows us to recover a series of contrast-weighted images which follow the signal dynamics given by the transient-state model. For example, one can observe contrast inversion between WM and GM as these signals evolve differently in time (see also Fig. 2b). (**c**) By design, combining frames of the reconstructed data creates signal hyperintensities which can be used to obtain vascular information, such as angiography projections (see Fig. 5). (**d**–**f**) Parameter inference techniques enable the computation of the voxel-wise parametric maps that best describe the signal evolutions. The entire 4D dataset of 112, 2 mm thick slices with 1.2 mm^2^ in-plane resolution was acquired in 6:48.

**Figure 5:**
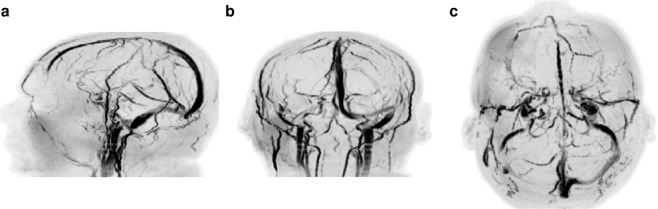
Angiography projections. Maximum intensity projections obtained by combining the last 160 frames of the reconstructed data (Fig. 4c) results in vasculature images. The angiography allows for an immediate visualization of all the main vascular structures in the head, such as the Circle of Willis, the carotid arteries, and the superior sagittal sinus. The projections show a left (**a**) and sagittal view, a coronal view (**c**), and an axial view (**d**). This information is directly obtained from the reconstructed measurements without the need for separate vascular scans.

**Figure 6:**
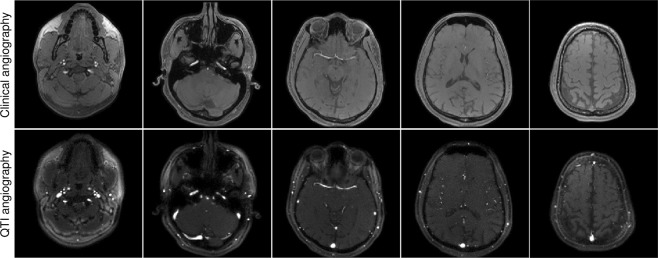
Validation versus standard clinical angiography. A comparison between the vascular information obtained from the reconstructed data shows a high degree of correspondence with a standard clinical time of flight angiography sequence, with the difference that QTI has increased sensitivity to slow blood flow in both arteries and veins. A maximum intensity projection over the data shown here produces the visualisations in Fig. 5, validating our approach as a novel method for creating imaging biomarkers from vascular information^70^.

We additionally validated our approach with volunteer data, where the obtained quantification proved consistent with literature reports on distinct tissue types in the brain^58–64^ (Supplementary Table 1). Figure 4 shows the results of the reconstructed data, from which we sum the absolute value of frames to obtain angiography images (Fig. 5) and make use of the signal dynamics of the reconstructed dataset to perform probabilistic parameter inference for the simultaneous estimation of PD, T1, and T2. Also, we compared the vasculature data obtained with QTI versus a standard clinical time of flight angiography pulse sequence, where the only notable difference is that our flow-resolved QTI application shows increased sensitivity to both slow and fast flowing blood in arteries and veins (Fig. 6, Supplementary Fig. 4 and Videos 1–2). It is important to note, however, that conventional time of flight angiography sequences are generally acquired at higher resolutions (0.86 mm^3^ isotropic in this example) than the QTI data acquired here with 1.2 mm × 1.2 × 2 mm resolution. We have re-scaled the QTI angiography data to make a direct comparison with the time of flight angiography possible. Notwithstanding, the simultaneous acquisition of this information could significantly impact the diagnosis of stroke and other vascular diseases. Importantly, for the patient and the radiologist these projections can be attained without additional specific angiography sequences at the expense of increased scan times: whereas conventional techniques would require serial measurements ranging from 30 minutes to one hour to obtain the same information, here the entire 4D set of complementary data is obtained in under seven minutes.

## Discussion

As MR sequences are developed towards multi-contrast, turnkey solutions, the methods described here offer a roadmap to systematically encode for additional parameters while simultaneously adding valuable image biomarkers derived from non-encoded parameters that affect the acquired signal. This was achieved by incorporating flow into the signal model, developing a design framework that accounts for signal contrast and parameter encoding, reconstructing space-resolved temporal signals, and performing a comprehensive analysis on these signals.

In theory, a quantification of the velocity of flowing blood is also possible with our proposed sequence. However, our sequence does not explicitly encode for velocity and has low sensitivity to its changes (Supplementary Fig. 1). Also, the constant velocity scalar used in our model oversimplifies the complex reality of flow inside vessels, leading to parametric information with unclear clinical utility (Supplementary Fig. 5). Moreover, the model accounts only for flow perpendicular to the slice, while failing to capture movement within the slice. Incorporating flow sensitive gradients in the design process and adjusting the model to account for more complex motion and perfusion phenomena is a possibility to extract flow information inherent in the data, and this is the subject of future work. On the other hand, this model, however simple, proved useful in the context of contrast maximisation: it provided vasculature images that could be immediately used in clinical routines. The creation of these images comes with no additional penalty on acquisition times; on the contrary, this is the fastest demonstration of transient-state parameter mapping – and the only capable of additionally obtaining relevant angiography images.

Several works have considered optimising sequences, either based on Cramér-Rao lower bounds or other metrics derived from the Fisher information matrix^14,65–67^, or by designing to maximise contrast for particular tissue classes^68^. Equation 8, conversely, considers both parameter encoding and contrast maximisation. Moreover, by using a prior, the optimization is not limited to a single sample of parameters or a restricted range specified from a training set. Rather, the design problem can be tackled in a multiclass, multiparametric fashion, ensuring the optimised design performs well for a wide range of values. Also, the information provided by the tissue priors can be used to incorporate additional design constraints, such as contrast maximisation of tissue classes. Thereafter, it is straightforward to extend this design framework to other uses by either modifying the prior to be e.g. specific to certain diseases, enforcing higher contrast between certain classes, or focusing exclusively on parameter encoding.

Both our Bayesian design framework and Bayesian parameter inference techniques operate under the simplifying assumption of a Gaussian noise model. Although these assumptions may be violated by combining complex data from multiple receiver coils and reconstructing using low rank approximations, we observed only a small model error with 10 subspace coefficients (Supplementary Fig. 6) and no significant differences when comparing against other inference techniques, such as dictionary matching or simple least squares fitting (Supplementary Fig. 7). Moreover, the uncertainty provided by Bayesian inference resulted in additional information regarding sequences encoding and parameter quantification. Also, the subspace images on themselves offer discriminative information between different classes (Supplementary Fig. 8), and could be used for subsequent image processing tasks, such as tissue segmentation.

## Conclusion

In this work, we have introduced a method to obtain complementary information from a single acquisition. The techniques proposed here account for motion phenomena during transient-state data acquisitions, present a sequence design framework that considers both contrast maximisation and parameter encoding, and offer a four-dimensional reconstruction to obtain spatially resolved temporal signals. From these reconstructed data we derive clinical imaging biomarkers in the form of angiograms and probabilistically infer the quantitative parameters of the Bloch equations. The information offered by our approach could provide valuable insight in automatically screening patients for stroke or stenosis, as vascular information can be directly obtained without the need of separate angiography scans.

Moreover, we consider adapting our technique to additional clinical biomarkers or quantitative parameters an interesting perspective for future research, as complementary data offers novel sources of information. Relying on the same design framework, it is conceivable to encode for more quantitative parameters, incorporate novel priors or different tissue classes – also disease-specific tissue classes – or emphasize unique tissue contrasts. This would open new dimensions that could deepen our understanding of the human body in health and disease.

## Supplementary information

Supplementary Material

Supplementary Video 1. QTI angiography versus clinical angiography video.

Supplementary Video 2. QTI angiography versus clinical angiography projection video.

## Data Availability

All additional data supporting the findings of this study are available within the paper and its Supplementary Information.

## Code Availability

The code used to simulate the transient-state signals, reconstruct multiple images, and estimate parametric maps is available here: https://bitbucket.org/pgomezd/qti. The source code for Π4U can be downloaded from the authors’ website: http://www.cse-lab.ethz.ch/software/Pi4U. A simulated phantom is available in the code repository. The phantom can be used to test the algorithms presented in this study.
