# Supplementary material for: Designing contrasts for rapid, simultaneous parameter quantification and flow visualization with quantitative transient-state imaging
Source: Sci Rep. 2019 Jun 11;9:8468. doi: 10.1038/s41598-019-44832-w (PMC6560213; doi:10.1038/s41598-019-44832-w)
Supplement: Supplementary file 1 — Supplementary Material [file 41598_2019_44832_MOESM1_ESM.pdf]

Designing contrasts for rapid, simultaneous parameter quantification and flow visualization with quantitative transient-state imaging

Pedro A. Gómez [1], Miguel Molina-Romero [1], Guido Buonincontri [3,4], Marion I. Menzel [2], Bjoern H. Menze [1]

[1] Technical University of Munich, Munich, Germany

[2] GE Healthcare, Munich, Germany

[3] Imago7 Foundation, Pisa, Italy

[4] IRCCS Stella Maris, Pisa, Italy

Supplementary Material

Supplementary Figures

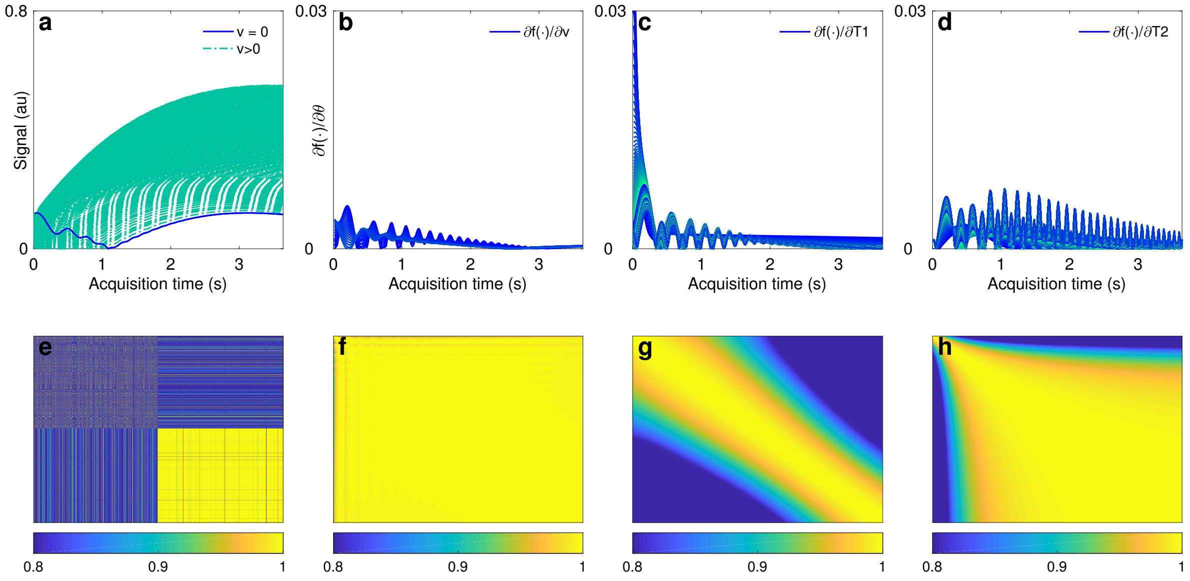

Supplementary Figure 1: Signal evolution and parameter sensitivity in the presence of flow. ***a****, After inversion, stationary blood (dark blue line) acts as a lower bound for flowing blood contrast. Even for slow flowing spins, non-stationary spins always have larger signal intensities than stationary spins.* ***b-d****, Gradient of the signal with respect to velocity (****b****), T1 (****c****), and T2 (****d****). The changes in velocity are comparatively small with respect to the changes in T1 or T2, signifying that the sequence is less sensitive to velocity changes.* ***e****, Correlation matrix from 2 x 10^3 samples in parameter space obtained by sampling their prior probabilities. The lower right quadrant represents areas where $v > 0$, indicating that signals in the presence of flow are highly correlated.* ***f-g,*** *Correlation matrices for continuously varied velocity (****f****), T1 (****g****), and T2 (****h****), where all other parameters are kept constant. Once again, a high correlation between signals containing velocity can be observed.*

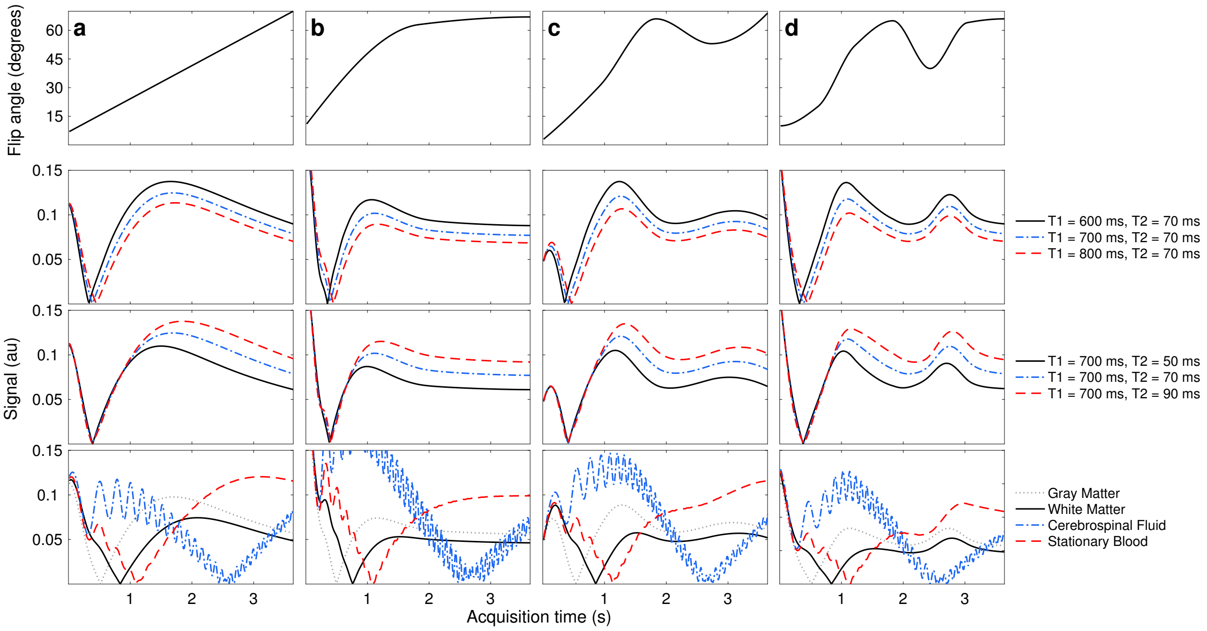

***Supplementary Figure 2: Alternative acquisition schemes. a**, Optimal acquisition scheme for two control points (initial and final flip angle) and corresponding transient-state signals for varying T1 values, varying T2 values, and the mean values of the tissue classes. **b-d**, The same design framework can be used to find higher order patterns with more control points. The optimal scheme is shown for three (**b**), five (**c**), and seven (**d**) control points, where the flip angle trajectory was determined with a smooth Hermite interpolating polynomial from one control point to the next.*

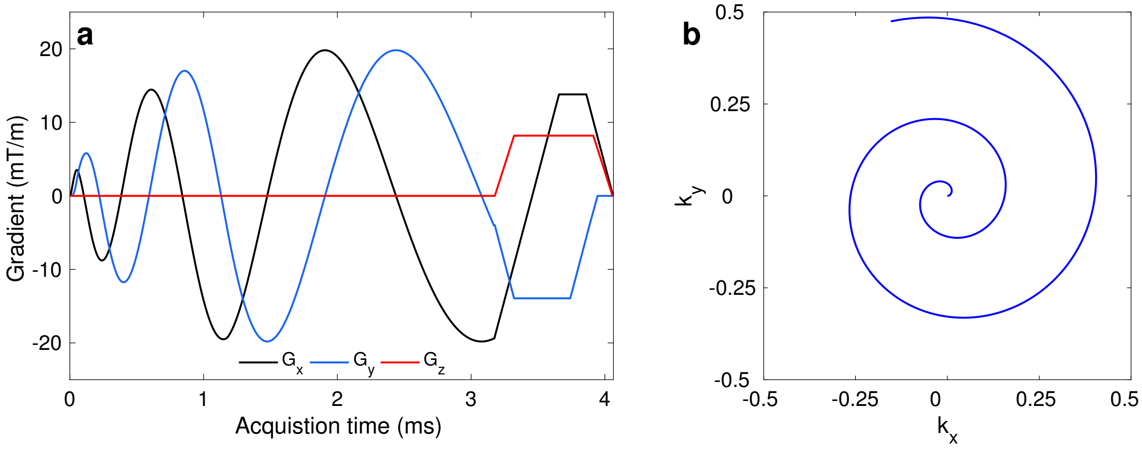

***Supplementary Figure 3. Imaging gradients**. **a**, A 4 ms spiral waveform is used in every repetition to acquire k-space data. As the x-y imaging gradients are rewound back to the centre of k-space, an unbalanced gradient in z leads to dephasing of the transversal magnetization. **b**, Acquisition trajectory of a single spiral interleave. This variable density spiral is rotated with the golden angle from one repetition to the next.*

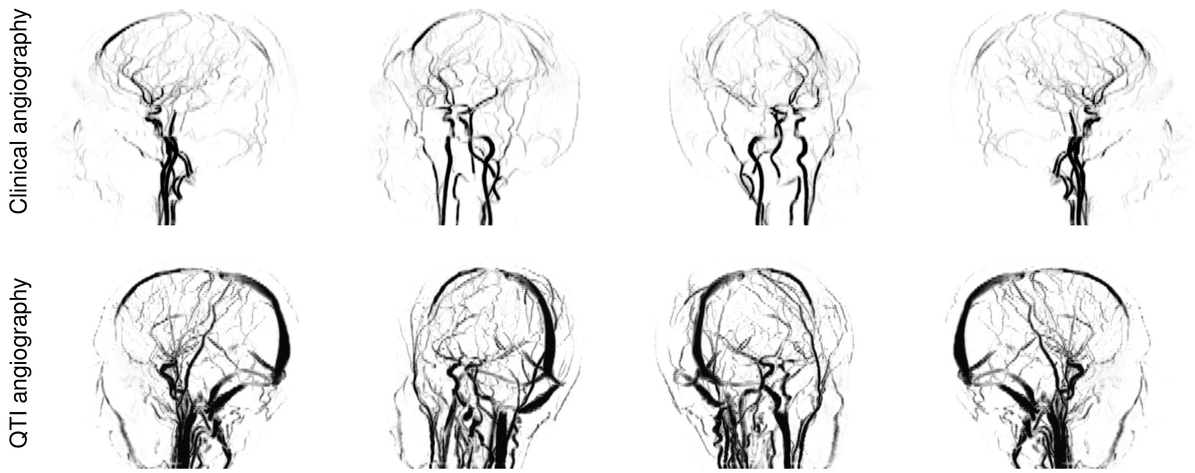

Supplementary Figure 4. Further validation versus clinical time of flight angiography. *The obtained angiography datasets for from the proposed acquisition are comparable to standard clinical time of flight angiography sequences. The images above show results from the same volunteer and orientations, where QTI is sensitive to slow and fast flowing blood in the arteries and veins. Supplementary Video 2 displays 360-degree rotations of these reconstructions.*

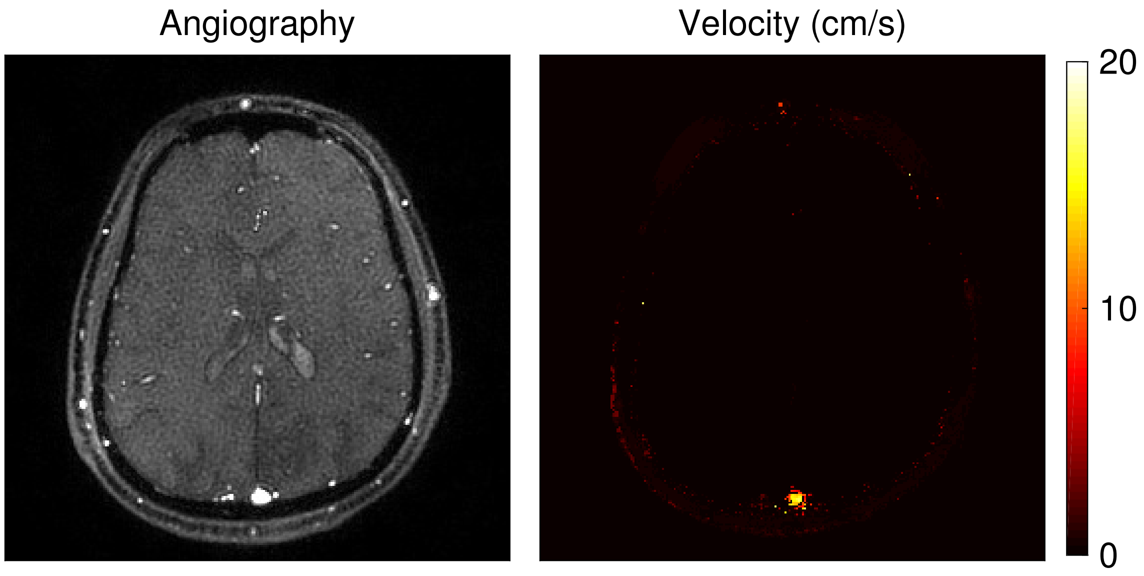

Supplementary Figure 5*. *Angiography versus velocity. *Whereas combining data from multiple frames results in vasculature information, it is also possible to use the reconstructed temporal signals to obtain a quantification of a velocity scalar. This velocity scalar is obtained by enforcing consistency with the signal model presented in Eq. 5 and appears prominently in large vessels, but fails to capture the small vessels visible in the vascular data. It is thus from the images, not the quantified parameters, that the maximum intensity projections for angiography can be obtained.*

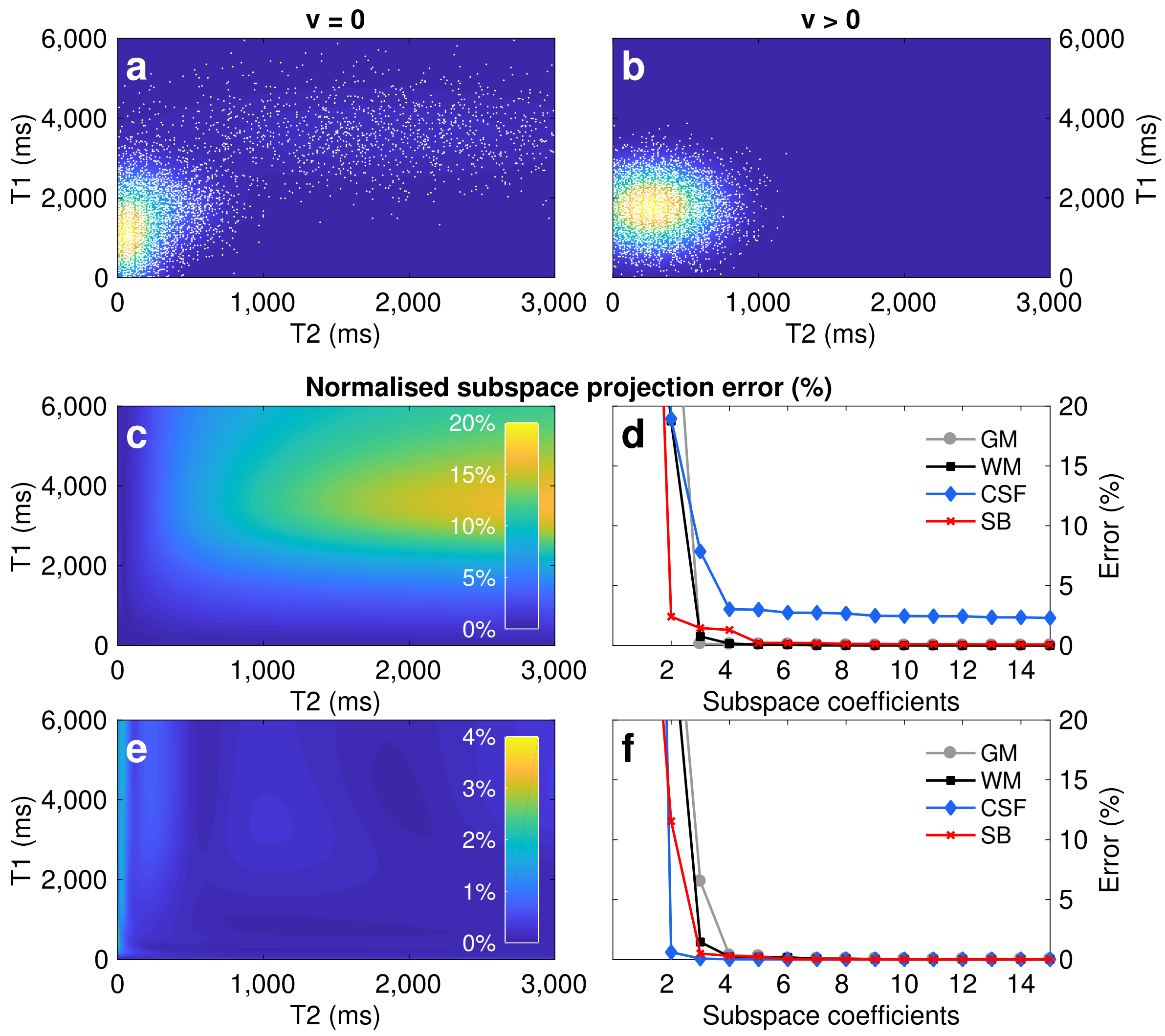

***Supplementary Figure 6. Prior sampling and normalized model error for subspace projection. a**, The four stationary tissue classes considered in this study were sampled according to their prior distribution. **b**, In the non-stationary case, only the blood prior was considered, whereas velocity was sampled evenly from 0 cm/s to 100 cm/s, which is the upper bound for spins remaining in the slice with an echo time of 2 ms and a slice thickness of 2 mm. **c**, Normalised subspace projection error for the parameter space and sparse prior sampling (1 x 10^3 samples) using ten subspace coefficients. The error increases in areas with less sampling, such as those with large T1/T2 values. **d**, Projection error as a function of temporal coefficients for each tissue class. With sparse sampling, the error for tissues with lower T1/T2 values becomes negligible*

after five subspace coefficients, whereas the error for CSF decreases to 3%, but does not approximate 0%. ***e****, Normalised subspace projection error for the parameter space and dense prior sampling (~1 x 10^6 samples) using 10 subspace coefficients. Here, the error remains below 4% over the entire parameter space.* ***f****, Projection error as a function of temporal coefficients for each tissue class and dense sampling. In this case, the error quickly decreases for all tissue classes, becoming negligible after five subspace coefficients.*

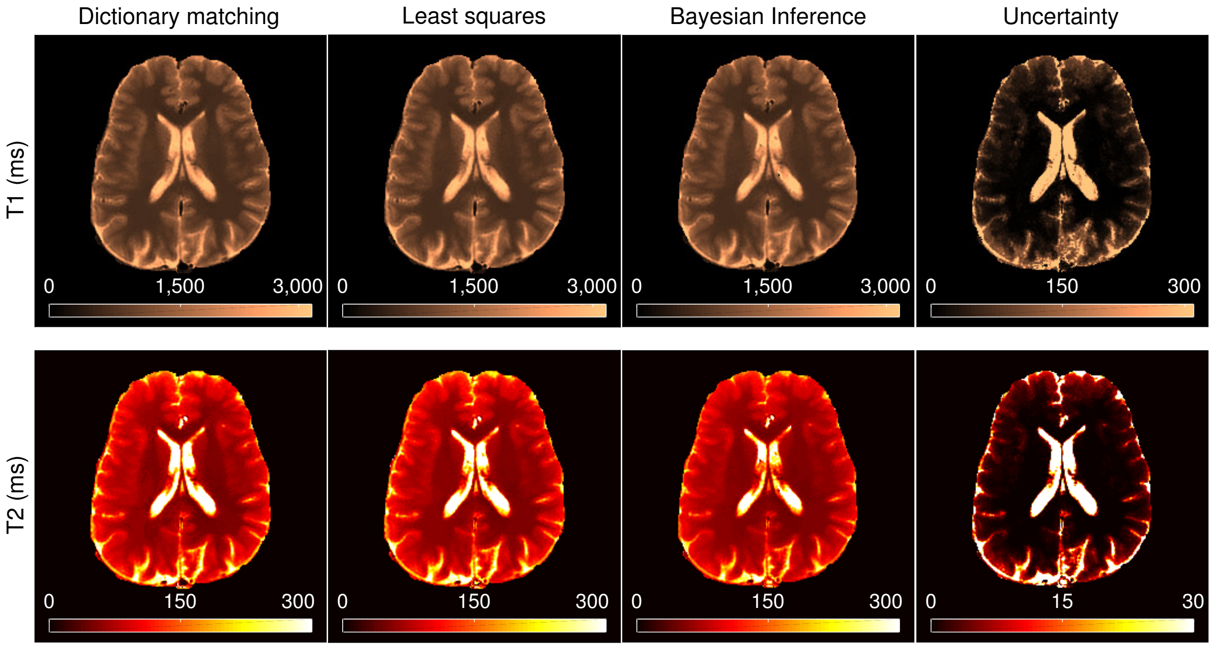

***Supplementary Figure 7*. *Comparison of different inference techniques*.** *Bayesian inference offers estimates of the parametric maps together with uncertainty quantification. While GM and WM show uncertainty below 5%, CSF had the highest uncertainty, reaching levels over 10% with respect to the estimated values. This increased uncertainty can be attributed to two factors. First, the sequence is designed to be more sensitive to lower T1/T2 values (such as GM and WM) instead of the large T1/T2 values present in CSF. Second, CSF has both diffuse and pulsatile motion, affecting parameter quantification and correspondingly increasing uncertainty. This additional feedback is not readily available with other parameter inference techniques, such as matching the reconstructed signals to a precomputed dictionary of performing a least-squares estimate of the parameters. Moreover, while these techniques are methodically different, we observed no systematic deviation from one to technique to the other.*

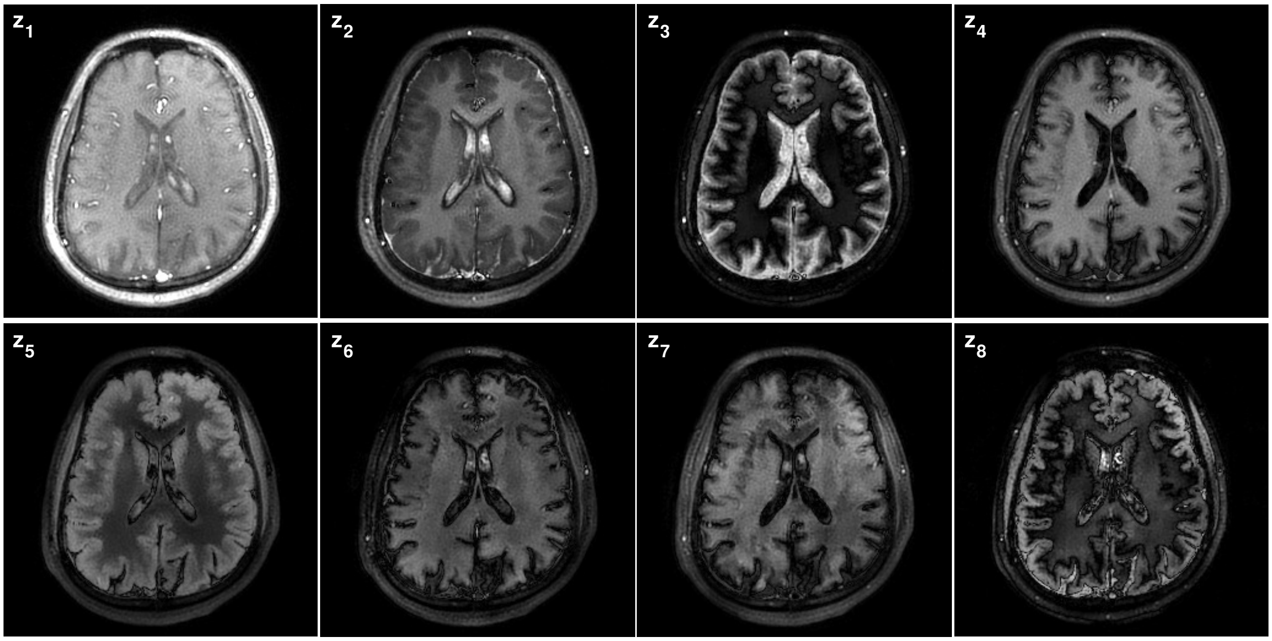

***Supplementary Figure 8**. **Subspace images.** The iterative reconstruction scheme proposed in this work recovers images in a lower dimensional temporal subspace. The angiography data, along with the different tissue classes, can also be observed in these images.*

Supplementary Videos

Supplementary Video 1. QTI angiography versus clinical angiography video. *The signal hyperintensities produced by flowing blood can be visualised in the entire volume for both the proposed reconstruction and a standard clinical time of flight angiography sequence. Image projections rely on these signal hyperintensities to create 3D visualisations of the vascular structures in the head (Fig. 5 and Supplementary Video 2).*

Supplementary Video 2. QTI angiography versus clinical angiography projection video. *A 360-degree rotation of the QTI angiography projections compared against the clinical angiography data shows the similarity between both techniques, validating our method as an alternative technique to obtain angiography information.*

Supplementary Tables

Supplementary Table 1: In vivo data compared to literature[1–7].

	T1 (ms)		T2 (ms)	
	**QTI**	**Literature**	**QTI**	**Literature**
**Grey matter**	1,248 ± 213	945 – 1,934	94 ± 20	61 – 106
**White matter**	799 ± 97	661 – 1,155	68 ± 12	52 – 72
**Cerebrospinal fluid**	4,078 ± 143	3,393 – 4,241	1,970 ± 408	1,000 – 2,500
**Blood**	1,593 ± 316	1,465 – 2,017	256 ± 85	225 – 325

Supplementary References

1. Lu, H. *et al.* Routine clinical brain MRI sequences for use at 3.0 tesla. *J. Magn. Reson. Imaging* **22,** 13–22 (2005).
2. Ethofer, T. *et al.* Comparison of Longitudinal Metabolite Relaxation Times in Different Regions of the Human Brain at 1.5 and 3 Tesla. *Magn. Reson. Med.* **50,** 1296–1301 (2003).
3. Gelman, N. *et al.* MR Imaging of Human Brain at 3.0 T: Preliminary Report on Transverse Relaxation Rates and Relation to Estimated Iron Content. *Radiology* **210,** 759–767 (1999).
4. MacKay, A. *et al.* Insights into brain microstructure from the T2 distribution. *Magn. Reson. Imaging* **24,** 515–525 (2006).
5. Smith, S. A., Edden, R. A. E., Farrell, J. A. D., Barker, P. B. & Van Zijl, P. C. M. Measurement of T1 and T2 in the cervical spinal cord at 3 Tesla. *Magn. Reson. Med.* **60,** 213–219 (2008).
6. Whittall, K. P. *et al.* In vivo measurement ofT2 distributions and water contents in normal human brain. *Magn. Reson. Med.* **37,** 34–43 (1997).
7. Noeske, R., Seifert, F., Rhein, K. H. & Rinneberg, H. Human cardiac imaging at 3 T using phased array coils. *Magn. Reson. Med.* **44,** 978–82 (2000).
